# What COVID-19 outbreak in Iran teaches us about virtual medical education

**DOI:** 10.1080/10872981.2020.1770567

**Published:** 2020-05-23

**Authors:** Kamran Aghakhani, Mohammadreza Shalbafan

**Affiliations:** a School of Medicine, Forensic Medicine, Iran University of Medical Sciences, Tehran, Iran; b Mental Health Research Center, Education and Development Office, School of Medicine, Iran University of Medical Sciences, Tehran, Iran

**Keywords:** Medical Education, medical students, COVID-19 pandemic, Iran

The first case of COVID-19 in Iran was officially reported by the Iranian government on February 19^th^ [1]. In order to control the rate of infection, the government decided to close all universities and schools to encourage people to stay home. As a consequence, the school of Medicine of Iran University of Medical of Sciences cancelled all classes not only at hospitals but also at university.

As no exact date could be estimated for the end of university shutdowns, we had to continue medical education through e-learning-based education, which is one of the best options to cope with this situation [2,3]. Therefore, all departments were asked and encouraged to upload their lessons on the university website and prepare new study material by using up-to-date facilities and tools provided before.

After several follow-ups, on the one hand, 616 multimedia educational materials were provided by departments of basic sciences, 2 months after the beginning of the outbreak, where 72 of 98 (73.5%) faculty members were involved. On the other hand, 523 materials were produced and uploaded by 227 of 541 (42%) faculty members of clinical departments during this time. Thus, the maintenance of both clinical and basic medical educations was satisfactory in general.

It is noticeable that departments of basic sciences could adjust in this new situation faster than departments of clinical sciences. In fact, even among the clinical departments, the ones that were not directly involved in the treatment of COVID-19 patients were able to make new adequate materials sooner, even though almost no study material was uploaded by the departments that had to deal with this disease the most in the first month of the outbreak.

In conclusion, virtual and e-learning-based medical education, including multimedia study materials, is extremely vital to provide an acceptable education for the undergraduate medical students during an outbreak. This is especially the case for a developing country like Iran, where most of the treatments all around the country are performed by academic hospitals and faculty members. So, it is plausible that during a medical crisis several departments, which are in the first line of treating the disease, cannot properly take part in this new style of medical education. Therefore, these departments would need more time to adapt to different means of teaching when social distancing is necessary.
